# Supplementary material for: HyperTRIBE uncovers increased MUSASHI-2 RNA binding activity and differential regulation in leukemic stem cells
Source: Nat Commun. 2020 Apr 24;11:2026. doi: 10.1038/s41467-020-15814-8 (PMC7181745; doi:10.1038/s41467-020-15814-8)
Supplement: Supplementary file 1 — Supplementary Information [file 41467_2020_15814_MOESM1_ESM.pdf]

Supplementary Information

HyperTRIBE uncovers increased MSI2 RNA binding activity and differential regulation in leukemic stem cells

Nguyen et al.

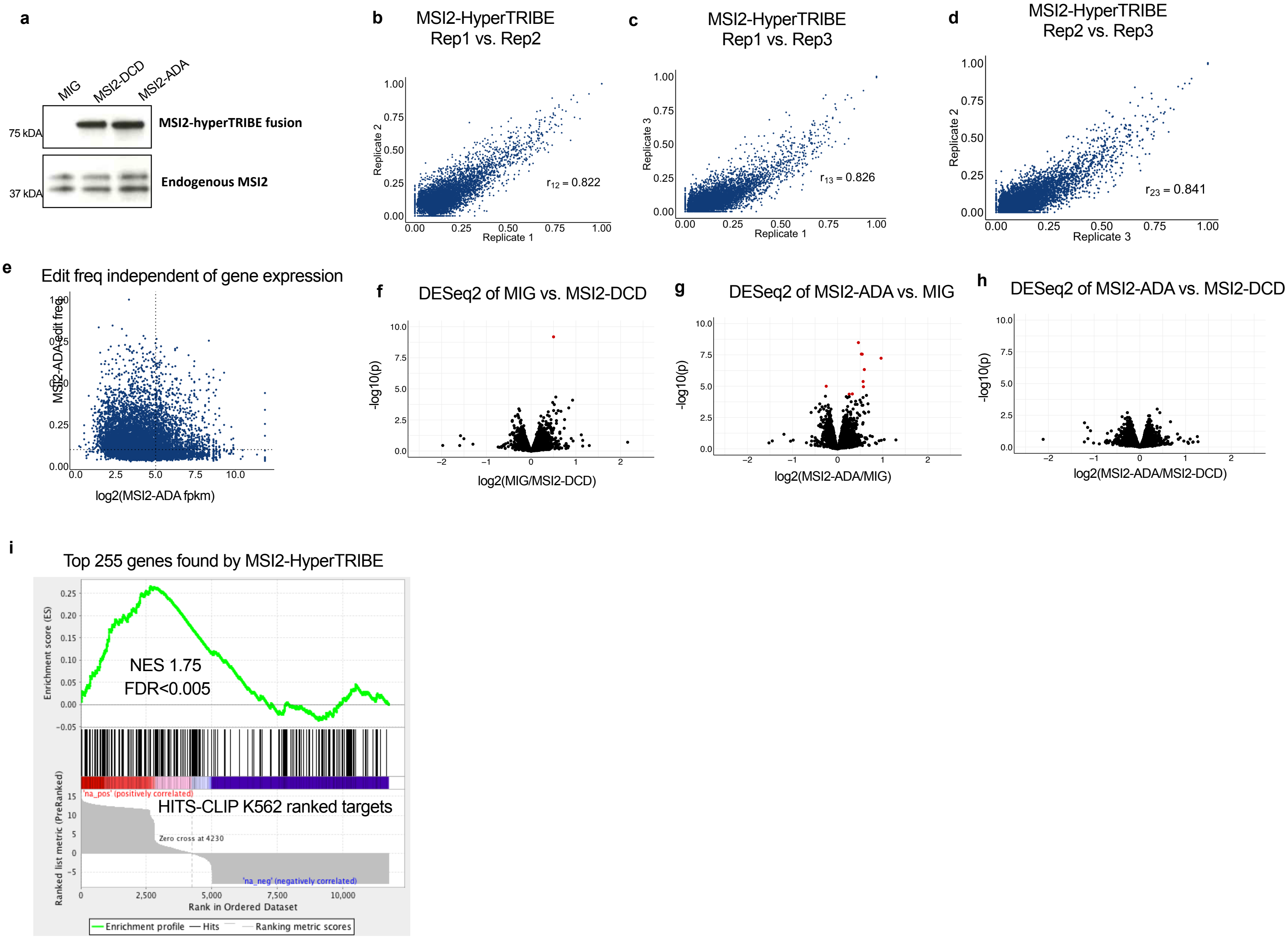

Supplementary Figure 1. Reproducible MSI2-HyperTRIBE editing and its independency from RNA target abundancy. a) Western blot analysis showing MSI2-ADA fusion protein and MSI2-DCD catalytic dead mutant control expression in MOLM-13 cells at 48hr post-transduction. b)–(d) Reproducibility of MSI2-HyperTRIBE in three independent experiments was tested by Pearson correlation (represented by R values) of the diff.frequency in pairwise experiments. e) Edit frequency in MSI2-ADA has no correlation with gene expression of edited RNAs, indicating that editing activity of MSI2-ADA is independent from the abundancy of mRNAs in the cells. f)–(h) Volcano plots of differential expression (DESeq2) between empty vector MIG control and MSI2-DCD expressing cells (f), between MSI2-ADA expressing cells and MIG control, and between MSI2-ADA expressing cells and DCD control. i) GSEA analysis was performed for top 255 gene targets with edit frequency of at least 0.4 and ranked list of target genes identified by HITS-CLIP in K562 cell line. Normalized Enrichment Score (NES) and FDR shows a positive correlation.

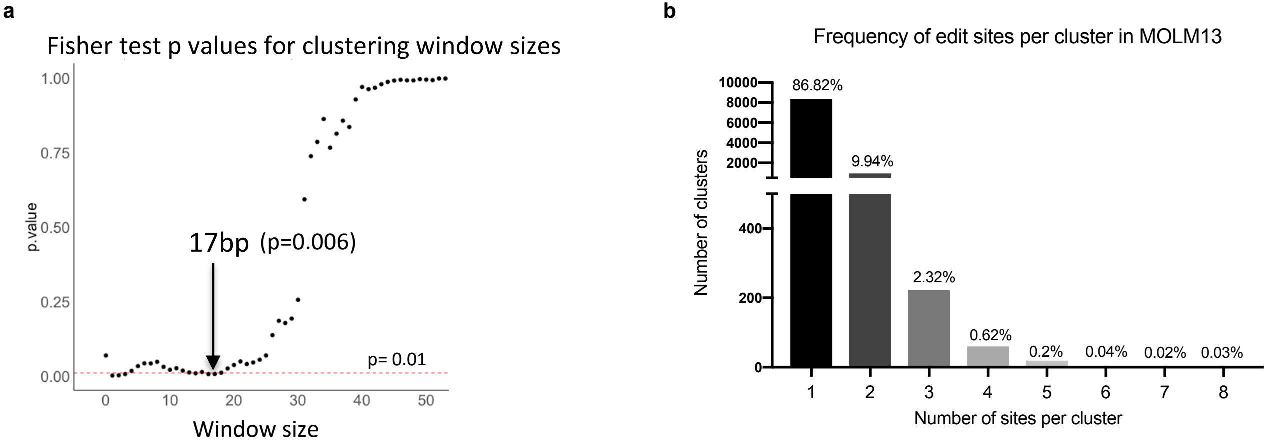

Supplementary Figure 2. MSI2-HyperTRIBE editing occurs at discrete sites. a) Fisher test determined that +/-17bp is the largest window such that the motif enrichment was significantly greater around true sites compared to background. b) Frequency of edit sites per cluster among all targets in MOLM13.

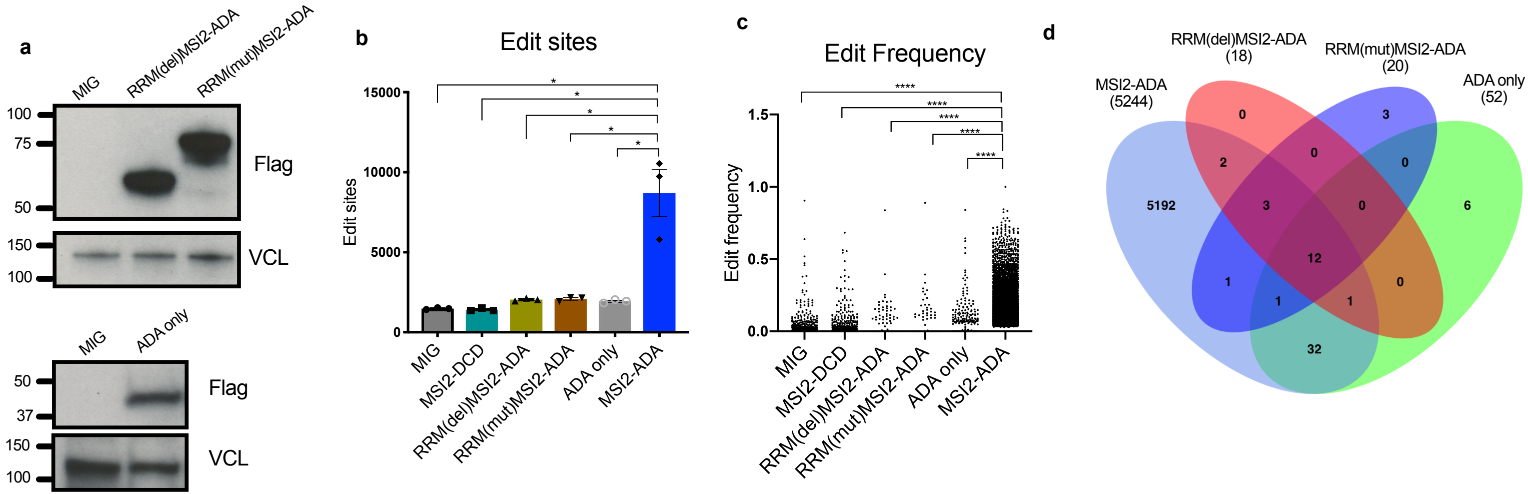

Supplementary Figure 3. MSI2-HyperTRIBE editing is dependent on its RRM domains. a) Immunoblot showing RRM(del)MSI2-ADA and RRM(mut)MSI2-ADA expression (upper panel); and ADAR catalytic domain alone (ADA only) (lower panel) compared to empty vector control (MIG). Vinculin (VCL) is a loading control. b) Number of edit sites in ADA only, RRM(del)MSI2-ADA and RRM(mut)MSI2-ADA compared to MSI2-ADA and controls MSI2-DCD and MIG. Data presented as mean +/- SEM from n=3 independent experiments. Two-tailed unpaired Student t-test; * $p < 0.05$. c) Comparing edit frequency by indicated constructs. Each data point is an edit site defined from three independent experiments (n=3). Data presented as mean +/- SEM. Unpaired Mann-Whitney test, **** $p < 0.0001$. d) Venn diagram overlapping significant edit sites by indicated constructs.

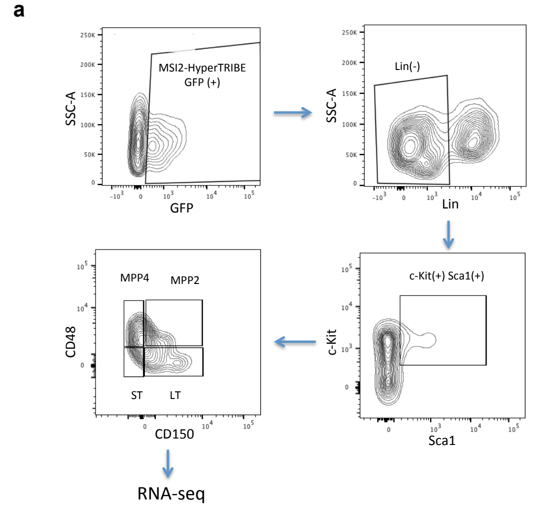

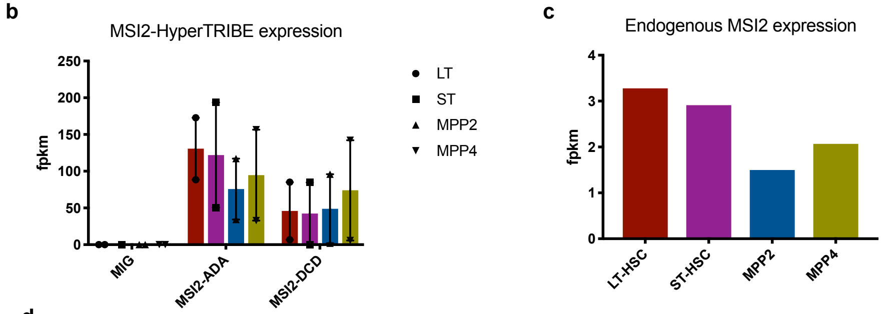

d

No. of Edit Sites	No. of Genes			
	LT	ST	MPP2	MPP4
1	625	585	528	547
2	155	133	96	82
3	41	43	23	19
4	19	11	6	10
5+	16	10	5	3

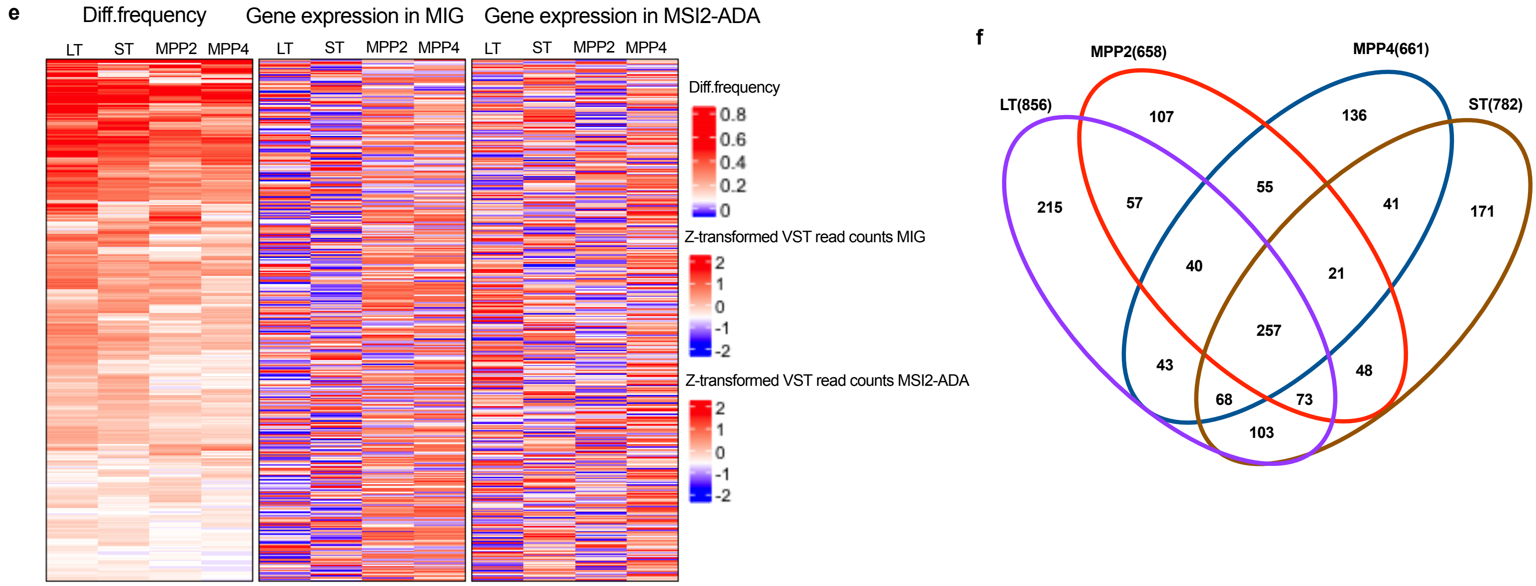

Supplementary Figure 4. MSI2-HyperTRIBE identifies RNA targets in HSPCs.
a) Sorting strategy for MSI2-HyperTRIBE in HSPCs. b) MSI2-ADA and MSI2-DCD fusion expression (RNA-seq data) in 4 HSPC populations. n= 2 independent experiments. c) Endogenous MSI2 expression in 4 HSPC populations (RNAseq). d) Table showing number of target genes with different bins of edit site numbers in 4 populations. e) Heatmap showing shared targets across all HSPC populations. These are the targets with no significant difference in diff.frequency across all populations. f) Venn Diagram overlapping MSI2 targets in 4 populations.

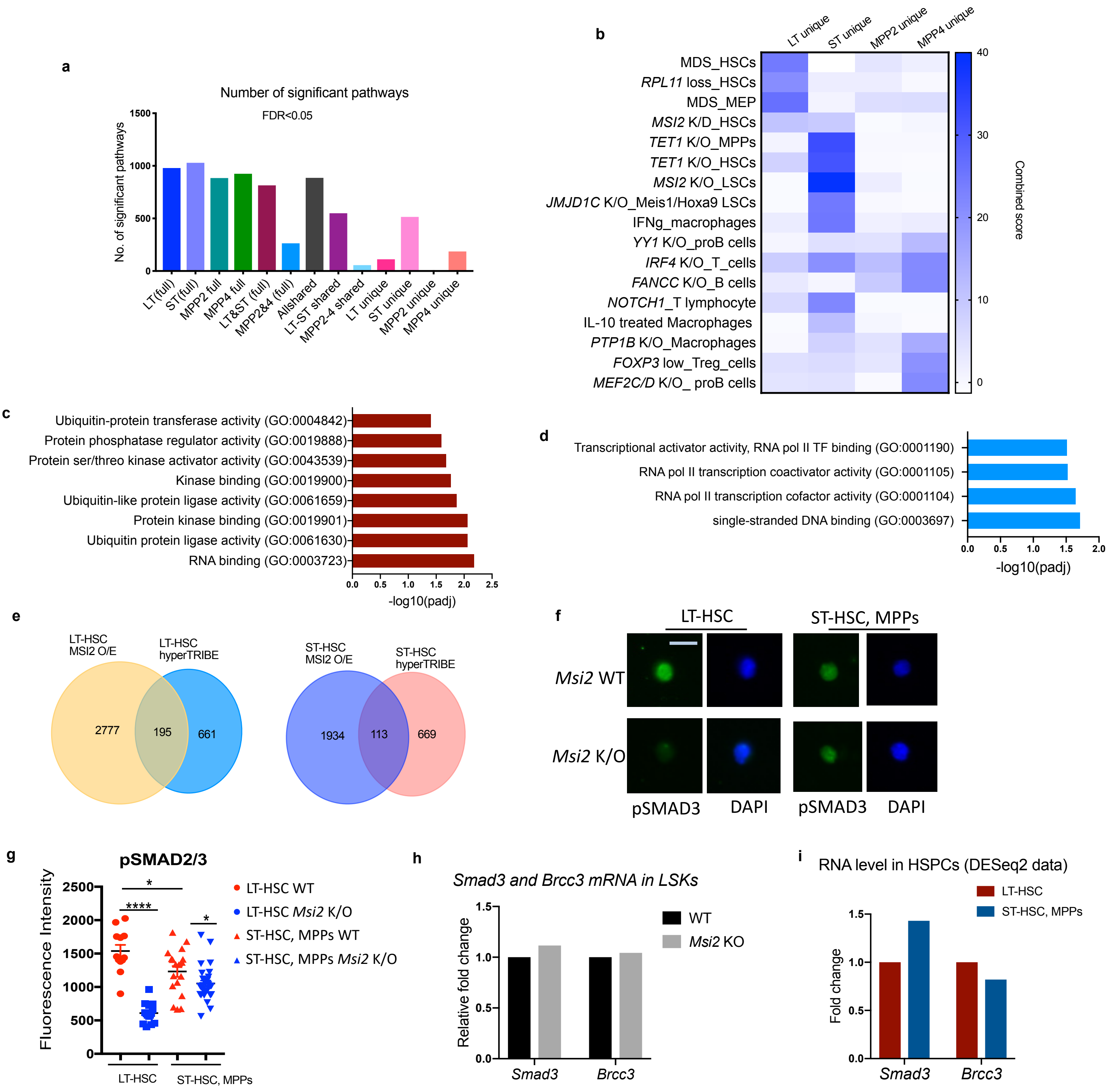

Supplementary Figure 5. MSI2 differential targeting program in HSPC subpopulations. a) Numbers of significant RNA-seq Gene & Drug pathways (FDR < 0.05) by ENRICHR analysis enriched in each of 4 populations (full), in LT&ST full (unique and shared targets in LT and ST populations), in MPP2&4 full (unique and shared targets in MPP2 and MPP4 populations), in All-shared (shared targets of all 4 populations), in LT&ST shared (shared targets in LT and ST), MPP2&4 shared (shared targets in MPP2 and MPP4) and in unique targets for each populations. Lists of these groups of targets are in Supplementary Table 3. b)Heatmap comparing score of top pathways enriched in unique targets of LT, ST, MPP2 and MPP4 populations. c) Gene Ontology (Molecular Functions) of LT and ST-HSC targets. Only significant pathways (adjusted *p*<0.05) were plotted (Full list in Supplementary Table 4). d) Gene Ontology (Molecular Functions) of MPP2 and MPP4 targets. Only significant pathways (adjusted *p*<0.05) were plotted (Full list in Table S4). e) Overlapping MSI2-HyperTRIBE targets and genes differentially expressed in MSI2 overexpression (MSI2-DCD) vs. control (MIG) in LT and ST-HSCs. f) Representative images of immunofluorescence analysis (IF) showing phosphorylated SMAD3 signal in LT-HSCs versus ST-HSCs and MPPs (MPP2 and MPP4) isolated from *Msi2* f/f Cre- (*Msi2* WT) and *Msi2* f/f Cre+ (Msi2 K/O). g) Quantitation of phosphorylated SMAD3 (pSMAD3) IF signal in LT or ST and MPPs after 4 weeks of pIpC treatment. n= 12; 15; 18; 31 cells for LT *Msi2* WT; KO; ST, MPPs WT and KO. Means +/- SEM. Student t-test. * $p < 0.05$, ****$p < 0.0001$. h) *Smad3 and Brcc3* mRNA level did not change upon *Msi2* KO in LSK (n=2 independent experiments. Microarray data) (Park et al., 2014). i) No significant difference in *Smad3* and *Brcc3* RNA abundance in LT-HSCs versus ST-HSCs and MPPs (RNAseq data in HSPCs in this study, n=2 independent experiments).

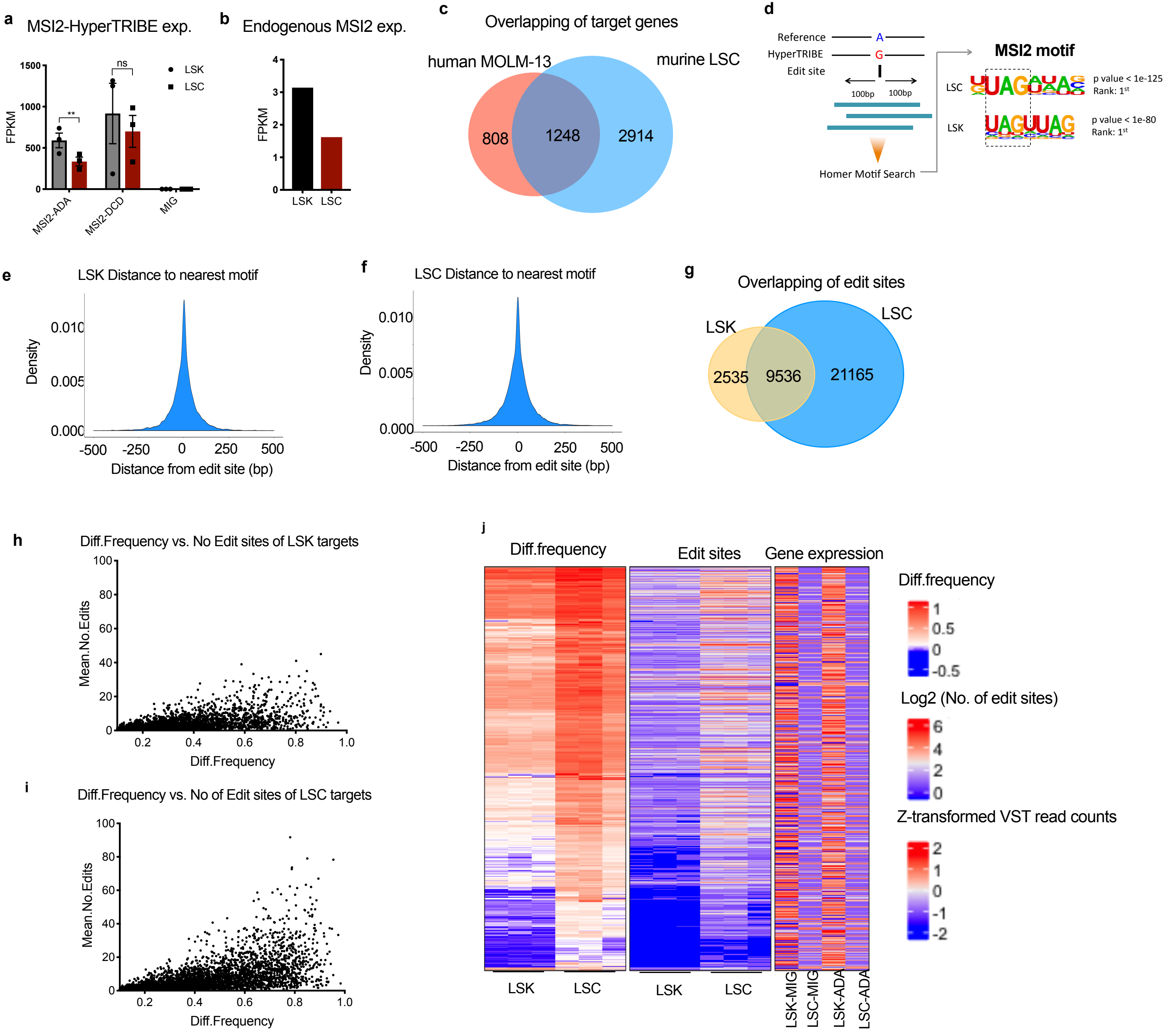

a
MSI2-HyperTRIBE exp.
FPKM
LSK
LSC
**
ns
MSI2-ADA
MSI2-DCD
MIG
b
Endogenous MSI2 exp.
FPKM
LSK
LSC
c
Overlapping of target genes
human MOLM-13
murine LSC
808
1248
2914
d
Reference
HyperTRIBE
Edit site
100bp
100bp
Homer Motif Search
MSI2 motif
LSC
p value < 1e-125
Rank: 1st
LSK
p value < 1e-80
Rank: 1st
e
LSK Distance to nearest motif
Density
Distance from edit site (bp)
f
LSC Distance to nearest motif
Density
Distance from edit site (bp)
g
Overlapping of edit sites
LSK
LSC
2535
9536
21165
h
Diff.Frequency vs. No Edit sites of LSK targets
Mean.No.Edits
Diff.Frequency
i
Diff.Frequency vs. No of Edit sites of LSC targets
Mean.No.Edits
Diff.Frequency
j
Diff.frequency
Edit sites
Gene expression
LSK
LSC
LSK
LSC
LSK-MIG
LSC-MIG
LSK-ADA
LSC-ADA
Diff.frequency
Log2 (No. of edit sites)
Z-transformed VST read counts

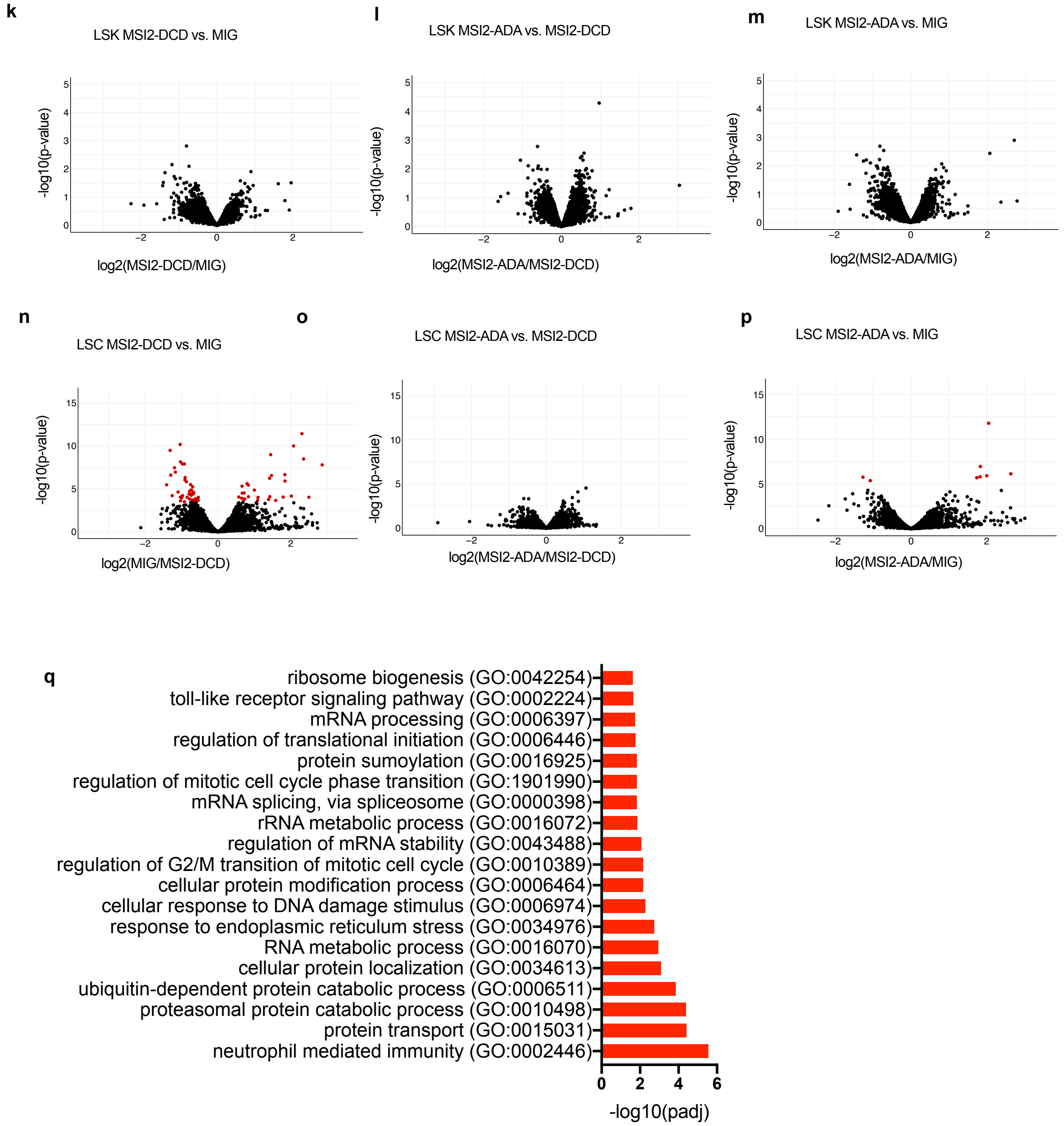

Supplementary Figure 6. Increased in MSI2 RNA binding activity in LSCs compared to LSKs.
a) MSI2-ADA and MSI2-DCD fusion expression in LSK and LSC cells. Data as means +/- SEM of FPKM (n=3 independent experiments). Significant difference in expression was evaluated using DESeq2 analysis. ** Adjusted $p < 0.01$. b) Expression level endogenous MSI2 in LSK and LSC cells, presented as FPKM in RNA-seq. c) Overlapping of MSI2 targets identified by HyperTRIBE in the human leukemia cell line MOLM-13 and murine leukemia stem cells (LSCs). d) *De novo* motif search showing MSI2 motif enrichment in LSKs and LSCs. e)- f) Probability Density Function (PDF) plots showing probability of MSI2 motif presence (shown in d) within a certain distance from an edit site in LSKs (e) and LSCs (f). g)Overlapping of significant edit sites in LSKs and LSCs. h) - i) Scatter plots showing number of edit site versus diff.frequency per target gene in LSKs (h) and LSCs (i). j) Clustering of diff.frequency by editing patterns for gene targets with a diff.frequency of at least 0.6 (for genes with multiple sites, the highest diff.frequency one is chosen) in LSKs or LSCs and fold change LSC fpkm/LSK fpkm <=1.2 (left panel). Matched number of edit sites for each target (per row) is shown in the middle panel and corresponding gene expression (Z-transformed VST-transformed read counts) in LSKs vs. LSCs, in empty vector cells MIG and in MSI2-ADA expressing cells (right panel). k) – m) Volcano plots of differential gene expression (DESeq2) in indicated LSKs. o) – p) Volcano plots of differential gene expression (DESeq2) in indicated LSCs. q) Significant pathways from Gene Ontology (Biological Functions) analysis enriched in LSC unique targets (Also in Supplementary Table 4).

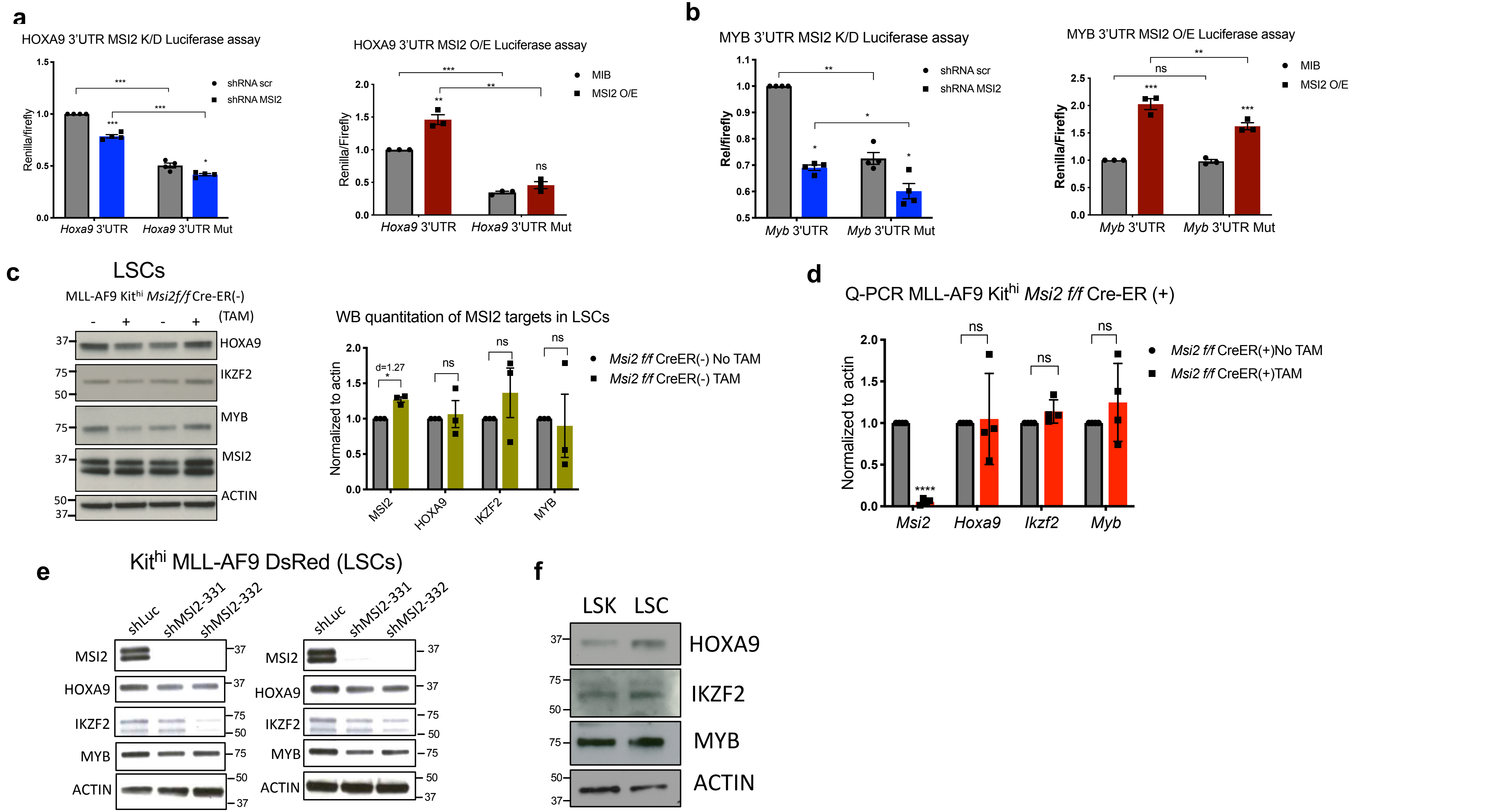

Supplementary Figure 7. MSI2 differentially controls Hoxa9, *Myb* and *Ikzf2* through binding sites in their 3'UTR in LSCs compared to LSKs.
a)- b) Luciferase reporter assay with original and mutated 3'UTR of *Hoxa9* (a) and *Myb* (b) in 293T cells. In mutated 3'UTR, all the MSI2 motifs found in LSKs and LSCs are mutated. shRNA scr: scrambled control, shRNA MSI2: shRNA specific to human *Msi2*, MIB: empty vector, MSI2 O/E: MIB containing human *Msi2* ORF. Data are presented as means +/- SEM of Renilla/Firefly ratio (n=3 for K/D experiments; n=4 for O/E experiments). Student t-test, *$p < 0.05$, ** $p < 0.01$, *** p < 0.001. c) Representative immunoblot images and quantitation of HOXA9, IKZF2 and MYB in *Msi2* Cre-ER (-) LSC controls. n= 3 independent experiments. Data as mean values +/- SEM. Paired Student t-test. d as the difference in the indicated bars. *p<0.05. d) Quantitative PCR showing unchanged mRNA level of *Hoxa9*, *Ikzf2* and *Myb* in *Msi2* Cre-ER(+) LSCs upon Msi2 depletion induced by Tamoxifen treatment. n=4 independent experiments. Data as mean values +/- SEM. ****p<0.0001. Paired Student t-test. e) Reduction of HOXA9, IKZF2 and MYB upon shRNA-mediated *Msi2* knock-down in MLL-AF9 Kit hi cells isolated from DsRed mice. Two independent experiments with similar results are shown. f) Immunoblot showing HOXA9, IKZF2 and MYB protein level in wildtype LSKs and LSCs. Three independent experiments with similar results are shown.

Supplementary Methods

Codon-optimised MSI2-ADA fusion sequence

ATGGAAGCCAACGGCAGCCAGGGCACCTCCGGCTCCGCCAATGACAGCCAACACGACCCCGGAAAGATGTTCATCGGCGGCCTCAGCTGGCAGA
CCAGCCCCGATAGCCTGAGGGACTACTTCTCCAAATTCGGCGAAATTAGGGAGTGCATGGTGATGAGAGACCCCACAACCAAGAGGTCCAGGGGCT
TCGGCTTTGTCACCTTCGCCGATCCCGCTTCCGTGGACAAGGTCCTGGGCCAGCCCCATCATGAGCTGGACTCCAAGACAATCGACCCCAAAGTGG
CCTTTCCCAGGAGAGCCCAGCCCAAGATGGTGACCAGGACCAAGAAGATCTTCGTGGGCGGCCTGAGCGCTAACACCGTGGTGGAGGACGTCAA
GCAATACTTCGAGCAGTTCGGAAAGGTCGAAGACGCCATGCTGATGTTCGACAAGACAACCAATAGGCACAGAGGCTTCGGCTTCGTCACCTTCGA
GAACGAGGATGTCGTCGAGAAGGTGTGCGAGATTCACTTCCACGAAATTAACAATAAGATGGTCGAGTGCAAGAAGGCTCAGCCTAAAGAGGTCATG
TTCCCCCCCGGCACAAGAGGCAGAGCTAGGGGCCTCCCCTATACAATGGACGCCTTCATGCTGGGCATGGGCATGCTGGGCTACCCCAACTTTGTG
GCTACATACGGCAGGGGCTATCCCGGCTTCGCCCCCTCCTATGGCTACCAGTTCCCCGGATTCCCCGCTGCTGCTTACGGACCCGTGGCCGCCGC
CGCCGTGGCTGCCGCCAGAGGCAGCGGATCCAATCCCGCTAGGCCTGGCGGCTTTCCCGGCGCTAATAGCCCTGGACCCGTCGCTGACCTGTATG
GACCCGCCTCCCAGGACAGCGGCGTGGGAAACTATATTTCCGCCGCTAGCCCCCAACCCGGATCCGGATTCGGCCATGGCATTGCCGGCCCCCTG
ATCGCTACCGCCTTCACCAATGGCTACCACTATTCCCCCATGGTGGTGCCCCAGAAGAACGTCCCCCTCCCCATTGACGACAAAAGCAGCAGCATGG
AGCTCCCCCAGATCCACGCTGACACAATTGGCAGGCTGGTGCTGGAAAAATTCATGGAGGTGATCAAAGGACAAGAGGCCTACAGCAGGAGGAAA
GTGCTGGCTGGCATTGTGATGACCGAGAATATGAACTTCTGCGAGGCCAAGGTGATCAGCGTGTCCACCGGCACCAAATGCGTCTCCGGCGAACAC
ATGAGCGTCAACGGCGCCGTGCTGAATGATTCCCACGCCGAGATTGTCTCCAGAAGGTGCCTCCTCAAGTACCTCTACGCCCAGCTGGACCTGCAG
TGCAACCAAGCTACCGCCTACCAGAGCATCTTCGTCAGAAACACCGATGGCCAGTACCCCTATAAGCTGAAGAGCGGAGTGCACTTCCACCTCTATA
TCAATACCGCTCCTTGCGGCGACGCTAGGATCTTCAGCCCTCACGAGAACGACACAGGCGTGGACAAGCATCCCAACAGGAAAGCCAGGGGCCAG
CTGAGGACCAAGATCGAGAGCGGCCAGGGAACCATCCCCGTGAAGAGCAGCGATGGCATCCAGACCTGGGATGGAGTCCTCCAGGGCCAGAGAC
TGCTGACCATGTCCTGTAGCGACAAGATCGCCAGGTGGAATATCGTGGGCATTCAAGGCTCCCTCCTGTCCAGCATCATTGAACCCGTGTACCTGCA
CTCCATCGTGCTGGGCTCCCTGCTGCACCCCGAACACATGTATAGGGCTGTGTGCGGCAGAATTGAGAAGAGCATCCAGGGCCTCCCTCCCCCCTA
CCACCTCAACAAACCCAGGCTGGCCCTCGTCACCAGCGCCGAGCCCAGAAACCAGGCCAAAGCCCCCAACTTCGGCATCAACTGGACCATCGGAG
ATACCGAGCTGGAGGTGGTGAACAGCCTCACCGGAAGGACCATTGGCGGCCAGGTGTCCAGGATCACAAAGCAGGCCTTCTTCGTGAAATACGGC
TTCCTCATGGCCAACCTGCCTGGCATCCTGGTGAGAAAAGTCACCACAGATTACGGCCAGACAAAAGCCAACGTGAAGGACTACCAGATTGCCAAG
CTGGAACTGTTCAGCGCTTTCAAGAGAGAAGACCTGGGCAGCTGGCTGAAGAAACCCATCGAACAGGATGAGTTCGGACTGGCTGAGGACTACAA
AGACGATGACGACAAGGATTATAAAGACGACGACGACAAGTGA

Codon-optimised MSI2-DCD fusion sequence

ATGGAAGCCAACGGCAGCCAGGGCACCTCCGGCTCCGCCAATGACAGCCAACACGACCCCGGAAAGATGTTCATCGGCGGCCTCAGCTGGCAGACCA
GCCCCGATAGCCTGAGGGACTACTTCTCCAAATTCGGCGAAATTAGGGAGTGCATGGTGATGAGAGACCCCACAACCAAGAGGTCCAGGGGCTTCGGC
TTTGTCACCTTCGCCGATCCCGCTTCCGTGGACAAGGTCCTGGGCCAGCCCCATCATGAGCTGGACTCCAAGACAATCGACCCCAAAGTGGCCTTTCC
CAGGAGAGCCCAGCCCAAGATGGTGACCAGGACCAAGAAGATCTTCGTGGGCGGCCTGAGCGCTAACACCGTGGTGGAGGACGTCAAGCAATACTTC
GAGCAGTTCGGAAAGGTCGAAGACGCCATGCTGATGTTCGACAAGACAACCAATAGGCACAGAGGCTTCGGCTTCGTCACCTTCGAGAACGAGGATGT
CGTCGAGAAGGTGTGCGAGATTCACTTCCACGAAATTAACAATAAGATGGTCGAGTGCAAGAAGGCTCAGCCTAAAGAGGTCATGTTCCCCCCCGGCAC
AAGAGGCAGAGCTAGGGGCCTCCCCTATACAATGGACGCCTTCATGCTGGGCATGGGCATGCTGGGCTACCCCAACTTTGTGGCTACATACGGCAGGG
GCTATCCCGGCTTCGCCCCCTCCTATGGCTACCAGTTCCCCGGATTCCCCGCTGCTGCTTACGGACCCGTGGCCGCCGCCGCCGTGGCTGCCGCCAG
AGGCAGCGGATCCAATCCCGCTAGGCCTGGCGGCTTTCCCGGCGCTAATAGCCCTGGACCCGTCGCTGACCTGTATGGACCCGCCTCCCAGGACAGC
GGCGTGGGAAACTATATTTCCGCCGCTAGCCCCCAACCCGGATCCGGATTCGGCCATGGCATTGCCGGCCCCCTGATCGCTACCGCCTTCACCAATGG
CTACCACTATTCCCCCATGGTGGTGCCCCAGAAGAACGTCCCCCTCCCCATTGACGACAAAAGCAGCAGCATGGAGCTCCCCCAGATCCACGCTGACA
CAATTGGCAGGCTGGTGCTGGAAAAATTCATGGAGGTGATCAAAGGACAAGAGGCCTACAGCAGGAGGAAAGTGCTGGCTGGCATTGTGATGACCGAG
AATATGAACTTCTGCGAGGCCAAGGTGATCAGCGTGTCCACCGGCACCAAATGCGTCTCCGGCGAACACATGAGCGTCAACGGCGCCGTGCTGAATGA
TTCCCACGCCGCGATTGTCTCCAGAAGGTGCCTCCTCAAGTACCTCTACGCCCAGCTGGACCTGCAGTGCAACCAAGCTACCGCCTACCAGAGCATCTT
CGTCAGAAACACCGATGGCCAGTACCCCTATAAGCTGAAGAGCGGAGTGCACTTCCACCTCTATATCAATACCGCTCCTTGCGGCGACGCTAGGATCTTC
AGCCCTCACGAGAACGACACAGGCGTGGACAAGCATCCCAACAGGAAAGCCAGGGGCCAGCTGAGGACCAAGATCGAGAGCGGCCAGGGAACCATC
CCCGTGAAGAGCAGCGATGGCATCCAGACCTGGGATGGAGTCCTCCAGGGCCAGAGACTGCTGACCATGTCCTGTAGCGACAAGATCGCCAGGTGGA
ATATCGTGGGCATTCAAGGCTCCCTCCTGTCCAGCATCATTGAACCCGTGTACCTGCACTCCATCGTGCTGGGCTCCCTGCTGCACCCCGAACACATGTA
TAGGGCTGTGTGCGGCAGAATTGAGAAGAGCATCCAGGGCCTCCCTCCCCCCTACCACCTCAACAAACCCAGGCTGGCCCTCGTCACCAGCGCCGAG
CCCAGAAACCAGGCCAAAGCCCCCAACTTCGGCATCAACTGGACCATCGGAGATACCGAGCTGGAGGTGGTGAACAGCCTCACCGGAAGGACCATTG
GCGGCCAGGTGTCCAGGATCACAAAGCAGGCCTTCTTCGTGAAATACGGCTTCCTCATGGCCAACCTGCCTGGCATCCTGGTGAGAAAAGTCACCACA
GATTACGGCCAGACAAAAGCCAACGTGAAGGACTACCAGATTGCCAAGCTGGAACTGTTCAGCGCTTTCAAGAGAGAAGACCTGGGCAGCTGGCTGAA
GAAACCCATCGAACAGGATGAGTTCGGACTGGCTGAGGACTACAAAGACGATGACGACAAGGATTATAAAGACGACGACGACAAGTGA
