# Supplementary material for: HyperTRIBE uncovers increased MUSASHI-2 RNA binding activity and differential regulation in leukemic stem cells
Source: Nat Commun. 2020 Apr 24;11:2026. doi: 10.1038/s41467-020-15814-8 (PMC7181745; doi:10.1038/s41467-020-15814-8)
Supplement: Supplementary file 3 — Description of Additional Supplementary Information [file 41467_2020_15814_MOESM3_ESM.pdf]

Description of Additional Supplementary Files

File Name: Supplementary Data 1
Description: Gene lists of targets all cell types.

File Name: Supplementary Data 2
Description: De novo motif analysis.

File Name: Supplementary Data 3
Description: Enrichr Gene pathway Enrichment analysis.

File Name: Supplementary Data 4
Description: 4 GO analysis from enrichR for HSPC LSK and LSC.
