# Supplementary material for: HyperTRIBE uncovers increased MUSASHI-2 RNA binding activity and differential regulation in leukemic stem cells
Source: Nat Commun. 2020 Apr 24;11:2026. doi: 10.1038/s41467-020-15814-8 (PMC7181745; doi:10.1038/s41467-020-15814-8)
Supplement: Supplementary file 8 — Reporting summary [file 41467_2020_15814_MOESM8_ESM.pdf]

nature research

Corresponding author(s): Michael Kharas

Last updated by author(s): Mar 10, 2020

Reporting Summary

Nature Research wishes to improve the reproducibility of the work that we publish. This form provides structure for consistency and transparency in reporting. For further information on Nature Research policies, see Authors & Referees and the Editorial Policy Checklist .

Statistics

For all statistical analyses, confirm that the following items are present in the figure legend, table legend, main text, or Methods section.

n/a	Confirmed	
☐	☒	The exact sample size (*n*) for each experimental group/condition, given as a discrete number and unit of measurement
☐	☒	A statement on whether measurements were taken from distinct samples or whether the same sample was measured repeatedly
☐	☒	The statistical test(s) used AND whether they are one- or two-sided<br>*Only common tests should be described solely by name; describe more complex techniques in the Methods section.*
☒	☐	A description of all covariates tested
☐	☒	A description of any assumptions or corrections, such as tests of normality and adjustment for multiple comparisons
☐	☒	A full description of the statistical parameters including central tendency (e.g. means) or other basic estimates (e.g. regression coefficient) AND variation (e.g. standard deviation) or associated estimates of uncertainty (e.g. confidence intervals)
☐	☒	For null hypothesis testing, the test statistic (e.g. *F*, *t*, *r*) with confidence intervals, effect sizes, degrees of freedom and *P* value noted<br>*Give P values as exact values whenever suitable.*
☒	☐	For Bayesian analysis, information on the choice of priors and Markov chain Monte Carlo settings
☒	☐	For hierarchical and complex designs, identification of the appropriate level for tests and full reporting of outcomes
☐	☒	Estimates of effect sizes (e.g. Cohen's *d*, Pearson's *r*), indicating how they were calculated

Our web collection on statistics for biologists contains articles on many of the points above.

Software and code

Policy information about availability of computer code

Data collection	All software used for data collection is commercially available and stated in the Methods section (with detailed information of version used). To remark, for cell sorting, BD FACS Aria II cell sorter instrument (November 2008 edition) and BD FACSDiva software (version 8.0.1 2014) were used. For immunofluorescence analysis, AxioVision Rel.4.8.2 (06-2010) software and Zeiss Imager Z2 (Zen 2 Blue Edition) were used. For beta binomial test analysis, VGAM (Version 1.1-2) and bbmle (Version 1.0.23.1) R packagefor bulk RNA-seq:Illumina HiSeq platform. For bulk RNA-seq:Illumina HiSeq platform was used.
Data analysis	For beta binomial test analysis, VGAM (Version 1.1-2) and bbmle (Version 1.0.23.1) R package were used. For bulk RNA-seq:Illumina HiSeq platform. ENRICHR 2013 (gene set pathway enrichment analysis, developed by Ma'ayan lab), GSEA v3.0 (Gene set pathway enrichment analysis), HOMER v4.10 (Motif analysis), STAR aligner 2.7 (read alignment method, Dobin et al., 2013), GATK (variant calling, Van der Auwera et al., 2013). Other custom code has been deposited into GitHub and can be accessed using SSH git@github.com:DiuTTNguyen/MSI2_HyperTRIBE_codes.git

For manuscripts utilizing custom algorithms or software that are central to the research but not yet described in published literature, software must be made available to editors/reviewers. We strongly encourage code deposition in a community repository (e.g. GitHub). See the Nature Research guidelines for submitting code & software for further information.

Data

Policy information about availability of data

All manuscripts must include a data availability statement. This statement should provide the following information, where applicable:

- Accession codes, unique identifiers, or web links for publicly available datasets
- A list of figures that have associated raw data
- A description of any restrictions on data availability

Accession codes, unique identifiers or web links are available throughout the manuscript or Methods section.All the RNA-seq data generated in this study have been deposited in the Gene Expression Omnibus database under the accession number GSE132949 and made available to the public.

Field-specific reporting

Please select the one below that is the best fit for your research. If you are not sure, read the appropriate sections before making your selection.

- [x] Life sciences
- [ ] Behavioural & social sciences
- [ ] Ecological, evolutionary & environmental sciences

For a reference copy of the document with all sections, see nature.com/documents/nr-reporting-summary-flat.pdf

Life sciences study design

All studies must disclose on these points even when the disclosure is negative.

Sample size	For in vitro HyperTRIBE in MOLM13, LSKs and LSCs, qPCR, Western blot and Luciferase reporter assay we aimed for a number of at least 3 independent experiments per group to allow basic statistical inference. For in vivo HyperTRIBE in HSPCs, LT-HSC, ST-HSC, MPP2 and MPP4 cells were sorted from at least 3 mice per group of MIG, MSI2-ADA and MSI2-DCD. Due to limited cell numbers of these stem and progenitor populations and materials required for RNA-seq, cells were pooled into 2 biological replicates for library prep and sequencing at the end.
Data exclusions	No data were excluded from the analysis with the exception of one MSI2 knock-out in MLL-AF9 MSI2 f/f RosaCre(+) experiment as the TAM did not work well and the depletion of MSI2 was not good. These exclusion criteria were pre-established.
Replication	All attempts at replication were successful. For reproducibility in HyperTRIBE trial in MOLM-13, we used Pearson correlation analysis and indicated in the manuscript that the editing activity of MSI2-HyperTRIBE is highly reproducible based on r correlation coefficient. The number of independent biological repeats performed for each experiment are indicated throughout the manuscript main content, methods and figure legends.
Randomization	We allocated recipient mice into different group randomly in transplant and in vivo experiments.
Blinding	No experiments were blinded.

Reporting for specific materials, systems and methods

We require information from authors about some types of materials, experimental systems and methods used in many studies. Here, indicate whether each material, system or method listed is relevant to your study. If you are not sure if a list item applies to your research, read the appropriate section before selecting a response.

Materials & experimental systems

n/a	Involved in the study	
[ ]	[x]	Antibodies
[ ]	[x]	Eukaryotic cell lines
[x]	[ ]	Palaeontology
[ ]	[x]	Animals and other organisms
[x]	[ ]	Human research participants
[x]	[ ]	Clinical data

Methods

n/a	Involved in the study	
[x]	[ ]	ChIP-seq
[ ]	[x]	Flow cytometry
[x]	[ ]	MRI-based neuroimaging

Antibodies

Antibodies used	For immunoblotting analysis, Msi2 antibody (Abcam, #76148), IKZF2 antibody (Santa Cruz, sc-9864, dilution 1:1000), MYB antibody (Millipore, 05-175, dilution 1:1000) and ACTB antibody (beta-actin-HRP, dilution 1:30000) (Sigma-Aldrich, A3854) were used. For immunofluorescence analysis, anti-SMAD3 (Cell Signaling Technology, 9523S, dilution 1:1000), anti-phosphorylated SMAD2/3 (Cell Signaling Technology, 8685S, dilution 1:1000) or anti-BRCC3 (Novus Biologicals, NBP1-76831, dilution 1:1000) were used at the dilution of 1:1000. For flow cytometry sorting, CD3 (Fisher #15-0031-83), B220 (ebioscience #15-0452-83), CD4 (Fisher #5013997), CD8 (ebioscience #15-0081-83), Gr-1 (ebioscience #15-5931-82), Ter119 (ebioscience #15-5921-83) (all conjugated with PE-Cy5), CD117-APC-Cy7 (Biolegend #105826), Sca-1-Pacific Blue (Biolegend #122520), CD150-APC (Biolegend #115910) and CD48-PE (Fisher #557485) (all from Invitrogen) were used with dilution of 1:200.
Validation	All antibodies have been validated for the indicated applications by the manufacturers.

Eukaryotic cell lines

Policy information about cell lines

Cell line source(s)	293T and MOLM-13 cell lines were obtained through American Type Culture Collection (ATCC).

Authentication	The cell line was authenticated and mycoplasma tested by Genetica DNA Lab - Cell Line Testing (www.celllineauthentication.com).
Mycoplasma contamination	Beside Genetica DNA Lab unique test, cells were routinely tested and confirmed negative for mycoplasma in house by using a Mycoplasma Test from Lonza Biosciences (#LT07-218) according to manufacturer's instructions.
Commonly misidentified lines (See ICLAC register)	No commonly misidentified lines were used in the study.

Animals and other organisms

Policy information about studies involving animals; ARRIVE guidelines recommended for reporting animal research

Laboratory animals	6-8 week old female C57BL/6 were used for the HyperTRIBE HSPCs in vivo experiments (lethally irradiated at 450rad twice before bone marrow transplantation). Quaternary 6-8 week old female MLL-AF9 leukemia Actin-DsRed mice were sacrificed when the leukemia became apparent.
Wild animals	The study did not involve wild animals
Field-collected samples	the study did not involve field-collected samples
Ethics oversight	All animal studies were performed on animal protocols approved by the Institutional Animal Care and Use Committee (IACUC) at Memorial Sloan Kettering Cancer Center.

Note that full information on the approval of the study protocol must also be provided in the manuscript.

Flow Cytometry

Plots

Confirm that:

- [x] The axis labels state the marker and fluorochrome used (e.g. CD4-FITC).
- [x] The axis scales are clearly visible. Include numbers along axes only for bottom left plot of group (a 'group' is an analysis of identical markers).
- [x] All plots are contour plots with outliers or pseudocolor plots.
- [x] A numerical value for number of cells or percentage (with statistics) is provided.

Methodology

Sample preparation	Mice were euthanasia by carbon dioxide. Bones from hind legs, forelegs and/or vertebrae were isolated. Bone marrow (BM) was extracted by crushing the bones using mortar and pestle in RPMI + 2% FBS. The suspension was filtered through a 70 µm mesh. All samples were centrifuged for 5 min at 1,500 rpm. Viable cell numbers were determined using the Trypan blue staining. MOLM-13 cells and Mouse BM cells were washed twice with PBS, then stained with antibody cocktails (see details in the Method section) in RPMI 2% FBS for 30 minutes on ice. After that, the cells were washed once in PBS and then resuspended in RPMI 2% FBS for Flow cytometry sorting.
Instrument	BD FACS Aria II cell sorter instrument (November 2008 edition) was used for cell sorting.
Software	BD FACSDiva software (version 8.0.1 2014) were used for gating.
Cell population abundance	The purity of sorted cells were detected via flow cytometer after sorting and samples with purity higher than 95% were used.
Gating strategy	Gating strategies is provided in the supplementary figures

- [x] Tick this box to confirm that a figure exemplifying the gating strategy is provided in the Supplementary Information.
